# Editorial for Special Issue on Ultra-Precision Machining of Difficult-to-Machine Materials

**DOI:** 10.3390/mi16091004

**Published:** 2025-08-30

**Authors:** Chen Li

**Affiliations:** 1 School of Mechatronics Engineering, Harbin Institute of Technology, Harbin 150001, China; lichen1992@hit.edu.cn; 2 Suzhou Research Institute of HIT, Suzhou 215104, China; 3 Zhengzhou Research Institute, Harbin Institute of Technology, Zhengzhou 450046, China

## 1. Introduction for Special Issue of Ultra-Precision Machining of Difficult-to-Machine Materials

Difficult-to-machine materials, such as semiconductors [1,2], laser crystals [3,4], engineering ceramics [5,6], optical glass [7,8], superalloys [9,10], and composite materials [11,12,13], have been widely used in aerospace, integrated circuits, and energy power due to their superior mechanical properties and stable chemical characteristics. For instance, gallium nitride crystals are currently recognized as the most promising third-generation semiconductor material [14], with widespread applications in the aerospace and new energy sectors. YAG laser crystals serve as the primary host material for multi-kilowatt solid-state lasers [15]. Silicon carbide ceramics exhibit excellent specific stiffness and hardness, making them an ideal candidate material for fabricating large-aperture spaceborne reflectors [16]. Silicon carbide fiber-reinforced silicon carbide ceramic matrix composites are the preferred material for the thermal structural components of aircraft engines [17].

For advanced applications, these materials must be shaped into smooth substrates with high surface integrity using precision and ultra-precision machining technologies. Traditional mechanical machining techniques primarily encompass grinding [18], lapping [19], polishing [20,21], turning [22], milling [23], drilling [24], boring, etc. In recent years, with the development of high-energy beams and electrochemical processing technologies, composite energy field machining has increasingly demonstrated its advantages in machining difficult-to-machine materials in an efficient and ultra-precise manner. Examples of such technologies include laser-assisted machining [25,26], ultrasonic vibration-assisted machining [27], plasma-assisted machining [28], electrochemical–mechanical composite machining [29], and ion beam machining [30]. However, these materials exhibit high brittleness, high hardness, anisotropic damage [31], and high elasticity [32], which present significant challenges for efficient machining. Severe surface and subsurface damage, along with serious cutting tool wear, are easily generated during the machining process, inevitably compromising the dimensional accuracy and service life of the components while increasing production costs [33,34,35].

Understanding the mechanical properties [36,37], revealing the damage evolution and material removal mechanism at micro- and nanoscales [38,39], exploring innovative machining technology [40,41], developing an innovative cutting tool [42,43], and optimizing machining process parameters [44,45] are of great significance to realize the high efficiency and precision machining of difficult-to-machine materials. This Special Issue, “Ultra-Precision Machining of Difficult-to-Machine Materials”, highlights recent research advancements in four key aspects within the machining field of difficult-to-machine materials, namely material removal mechanisms [46], abrasive machining technology [46,47,48], composite energy field machining technologies [49,50,51,52,53], and the development of high-performance cutting tools [54]. These advanced theories and technologies offer significant novel insights into efficient and low-damage machining of difficult-to-machine materials, comprising nine original articles.

## 2. Overview of Published Articles

### 2.1. Material Removal Mechanism Induced by Machining of Difficult-to-Machine Materials

Elucidating damage evolution and material removal mechanisms is essential for achieving the efficient and precise machining of difficult-to-machine materials. Jahnel et al. [46] investigated the brittle-to-ductile transition and the material removal mechanisms of glassy carbon induced by abrasive machining through nano-scratching and indentation tests. The results indicated that glassy carbon exhibited a distinct brittle-to-ductile transition behavior during the nanoscratching process, and the substrate underwent successive stages of ductile plastic deformation, funnel-shaped fracture, and brittle conchoidal fracture as the cutting depth increased. Appropriately reducing the feed rate and grinding depth while using abrasives with finer grain size is beneficial for improving the workpiece’s surface quality. Inducing compressive stress during the machining process promotes more ductile material removal behavior due to the suppression of crack initiation and propagation. These findings not only enhance the understanding of material damage evolution and removal mechanisms at micro- and nanoscales but also provide a theoretical basis for achieving the high-efficiency and low-damage machining of brittle solids.

### 2.2. Abrasive Machining Technology of Difficult-to-Machine Materials

Abrasive machining technology involves the use of fine-grained abrasives or grinding wheels for workpiece precision machining. This technique represents a key method for achieving nanoscale ultra-smooth surface finishing of difficult-to-machine materials and primarily includes processes such as grinding, polishing, lapping, abrasive jet machining, and wire saw cutting. Flexible rheological polishing facilitates the fabrication of the smooth curved surfaces of GCr15 bushings. However, the inherent energy dissipation of the medium during flow leads to an uneven distribution of material removal. Zhao et al. [47] proposed controlling the physical field distribution of the abrasive medium through tool motion to achieve regulated material removal. In their study, the non-Newtonian behavior of the abrasive medium was characterized using a power law model. Based on principles of fluid dynamics and tribology, a film thickness model on the workpiece surface was developed. By solving this model, the pressure and velocity distributions within the film were determined. Based on contact mechanics, a single-abrasive-material removal model was developed, and a statistical method was employed to establish an abrasive distribution model, thereby enabling the formulation of a theoretical material removal model. Zhao refined this model through experimental validation, achieving a maximum error of no more than 14.0% and an average error of 11.1%. Guided by the corrected model, polishing a cylindrical inner surface for 60 min resulted in a surface roughness of 17.59 nm with a variance of 4.42 nm^2^.

Zhang et al. [48] investigated the influence of diamond wire saw processing parameters on the surface characteristics of silicon nitride ceramics. Sawing experiments on silicon nitride ceramics were conducted within the range of processing parameters for diamond wire sawing, and the effects of cutting parameters on the surface morphology, surface roughness, and waviness of the as-sawn slices were analyzed. The results indicated that material removal on the diamond wire as-sawn surface of silicon nitride ceramics predominantly occurred in a brittle mode, as evidenced by the presence of brittle pits and regularly distributed wire marks within the 20–55 µm scale range. The surface roughness of the slices along the workpiece feed direction ranged from 0.27 to 0.38 µm and decreased with increasing wire speed and decreasing feed rate. Surface waviness varied between 0.09 and 0.21 µm, showing a consistent trend with the variation in sliced-surface roughness. The study results offer an experimental basis for advancing the engineering application of diamond wire sawing technology in machining silicon nitride ceramic components.

### 2.3. Composite Energy Field Machining Technology of Difficult-to-Machine Materials

Compared with traditional machining technologies, high-energy beam and electrochemical machining methods can effectively reduce machining forces, residual stresses, and tool wear, thereby demonstrating their advantages in the efficient and ultra-precision machining of difficult-to-machine materials. Numerous researchers have investigated the material removal mechanisms and process optimization strategies in composite energy field machining for such materials. Zhang et al. [49] proposed a longitudinal–torsional ultrasonic grinding (LTUG) process for machining the inner surface of GCr15 bushings. Modal analysis and amplitude testing were conducted to verify the structural rationality of the LTUG setup. Based on the probability density function of cutting thickness and the overlapping effect of adjacent abrasive trajectories, a surface topography prediction model for LTUG was established using the height formula of the surface residual material. The model’s reliability was validated through orthogonal testing, achieving a prediction accuracy within 13.2%. According to the response surface methodology, the optimal process parameters were selected to satisfy the requirements of low surface roughness (Ra) and high material removal rate (MRR). The inner surface of the raceway plays a critical role in determining the performance of the bearing, and this study provides theoretical guidance for the longitudinal–torsional ultrasonic grinding process applied to the inner surface of GCr15 bearing steel.

Lin et al. [50] investigated the various fiber removal modes induced by orthogonal fiber weaving in SiCf/SiC composites. Force variations were analyzed through ultrasonic vibration-assisted scratching experiments to elucidate the influence of material removal mechanisms. In the study, three distinct surfaces, characterized by differences in fiber bundle weaving and lamination structures, were selected for comparative analysis. The results indicated that the internal fiber arrangement within the composite significantly affected the forces generated during the material removal process. Meanwhile, while investigating the influence of processing parameters on scratching forces, it was determined that the feed rate exerted the most significant effect. A comparative analysis between ultrasonic vibration-assisted scratching and traditional scratching experiments demonstrated that ultrasonic vibration can effectively reduce the scratching force. The underlying mechanism can be explained as follows: under the influence of ultrasonic assistance, the fibers experience brittle fracture, the matrix undergoes tearing, and surface residues are effectively removed in a timely manner. These combined effects contribute to a significant improvement in surface roughness after ultrasonic vibration-assisted processing.

Yang et al. [51] developed a novel anode vibration-assisted helical electrode electrochemical drilling method for fabricating micro-hole arrays on metal tube sidewalls in a highly efficient manner. Through comprehensive simulations and experimental investigations, the research team systematically examined the effects of the helical electrode rotation direction, rotational speed, and workpiece vibration parameters. The optimal results were obtained using forward electrode rotation at 3000 rpm in combination with workpiece vibration (8 μm amplitude at 100 Hz frequency), yielding precision micro-holes with a 200 μm diameter and a standard deviation of 3 μm. By employing this optimized process, the researchers successfully fabricated an array of 10 uniformly distributed micro-holes on 304 stainless steel tubes. Practical application tests demonstrated that the developed tube electrode enabled radial electrolyte flushing in electrochemical cutting, resulting in high-precision slits with an average width of 1.089 mm (standard deviation of 35.3 μm) on 5 mm thick stainless-steel plates. This breakthrough technology overcomes the critical limitations of conventional methods—such as drilling, laser cutting, or electrical discharge machining (EDM)—including thermal damage and burr formation, thereby offering an innovative solution for the efficient and precise machining of large-scale ruled surface components.

Cheng et al. [52] elucidated the mechanism underlying trajectory deviation and the uneven distribution of fracture quality in ceramic cutting using the thermal-controlled fracture method. Experimental results obtained from single-surface heating mode cutting demonstrate that the fracture trajectories on the upper and lower surfaces exhibit significant inconsistency, with the fracture quality being inferior to that achieved under dual-surface heating conditions. A finite element model was employed to calculate the stress distribution during the process, aiming to elucidate the underlying causes of the processing quality issues. This study demonstrates that trajectory deviation primarily results from the combined effects of lateral shear stress and transverse shear stress during ceramic cutting with a surface heat source. The uneven distribution of fracture quality in the single-surface heating mode is primarily attributed to the monotonic and highly gradient distribution of transverse tensile stress along the thickness direction of the workpiece. This study contributes to a deeper understanding of the processing challenges associated with this method, thereby facilitating the development of high-quality processing techniques in this field.

Wu et al. [53] conducted an investigation into quality control and damage mechanisms in the laser cutting of single-crystal silicon, systematically examining the regulatory effects of laser process parameters on cut seam dimensions and influenced processing zones in order to address the requirements of efficient wafer cutting. This study employed nanosecond pulsed laser cutting to process single-crystal silicon and innovatively applied surface erasure technology to eliminate the heat-affected zone. The results indicated that wiping the cutting surface effectively removed the processing-affected zones and recast layers on both sides of the cut seam, restoring the surface to a condition closely resembling its original state. Variations in the number of cutting passes influenced the surface morphology of the groove, with the optimal surface morphology observed at 20 cuts. This study quantitatively analyzed the correlation between laser parameters and damage morphology, demonstrating that optimized parameter settings can simultaneously achieve high cutting quality and minimal thermal damage, thereby offering a novel process approach for semiconductor manufacturing.

### 2.4. Development of High-Performance Cutting Tools

Cutting tools are often referred to as the “industrial teeth” of machine tools, and their performance directly influences production efficiency and machining quality. With the growing demand for high-precision and high-efficiency machining in sectors such as aerospace, increasingly stringent requirements have been placed on the performance of cutting tools. The development of technologies for fabricating high-performance cutting tools plays a crucial role in achieving the efficient and precise machining of difficult-to-cut materials. Qin et al. [54] proposed a chemical–mechanical synergistic preparation (CMSP) method for the cutting edge of cemented carbide inserts. A CMSP device was specifically designed and constructed to process insert cutting edges. Subsequently, the Taguchi method was integrated with gray relational analysis and fuzzy inference to optimize the polishing slurry formulation used in the CMSP process for insert cutting edges. In addition, orthogonal experiments, the Taguchi method, and analysis of variance (ANOVA) were employed to evaluate the effects of the polishing plate’s rotational speed, swing angle, and controller input frequency on the edge preparation process and to optimize the corresponding parameters. The results indicated that the optimal parameter combination for the polishing slurry used in the processing of cemented carbide inserts included an abrasive particle mass concentration of 10 wt%, an oxidant mass concentration of 10 wt%, a dispersant mass concentration of 2 wt%, and a pH value of 8. For the linear edge CMSP process, the optimal parameter combination consisted of a polishing plate rotational speed of 90 rpm, a swing angle of 6°, and a controller input frequency of 5000 Hz. The optimal CMSP process parameter combination for the circular edge included a polishing plate rotational speed of 90 rpm, a swing angle of 6°, and a controller input frequency of 7000 Hz. Among these parameters, the polishing plate rotational speed exerted the most significant influence on the edge preparation process, followed by the swing angle, while the controller input frequency had the least impact. This study demonstrated that CMSP represents a promising approach for treating cemented carbide insert cutting edges in tool manufacturing enterprises.

## 3. Conclusions

The articles published in this Special Issue focus on the precision machining of representative difficult-to-machine materials, including glassy carbon [46], high-strength steel [47,49,51], engineering ceramics [48,52], composite materials [50], monocrystalline silicon [53], and cemented carbide [54]. Authors have proposed numerous innovative machining technologies and process optimizations for the aforementioned difficult-to-machine materials, achieving significant results in material removal mechanisms, polishing, diamond wire saw cutting, ultrasonic vibration-assisted machining, laser-assisted machining, vibration-assisted electrode electrochemical drilling, and the fabrication of high-performance cutting tools. These research findings effectively improved the surface quality of the workpiece and machining efficiency while reducing cutting forces and tool wear. These achievements laid a theoretical foundation and provided technical support for developing efficient and low-damage machining technologies for difficult-to-machine materials.
